# Serious adverse events with novel beta-lactam/beta-lactamase inhibitor combinations: a large-scale pharmacovigilance analysis

**DOI:** 10.1007/s10096-020-04149-3

**Published:** 2021-01-07

**Authors:** Milo Gatti, Emanuel Raschi, Fabrizio De Ponti

**Affiliations:** Pharmacology Unit, Department of Medical and Surgical Sciences, Alma Mater Studiorum, University of Bologna, Via Irnerio 48, 40126 Bologna, Italy

**Keywords:** Ceftolozane-tazobactam, Ceftazidime-avibactam, Safety profile, Agranulocytosis, Neurotoxicity

## Abstract

The purpose of this study is to characterize adverse events (AEs) of clinical interest reported with ceftolozane-tazobactam and ceftazidime-avibactam, as an aid in monitoring patients affected by severe multidrug-resistant Gram-negative infections. We queried the worldwide FDA Adverse Event Reporting System (FAERS) and performed disproportionality analysis, selecting only designated medical events (DMEs) where ceftolozane-tazobactam and ceftazidime-avibactam were reported as suspect. Serious neurological AEs were further investigated. The reporting odds ratios were calculated, deemed significant by the lower limit of the 95% confidence interval (LL95% CI) > 1. All other drugs/events recorded in FAERS and cephalosporins showing clinical evidence of neurological AEs were respectively selected as comparator for analysis of DMEs and neurotoxicity. Qualitative analysis including case-by-case assessment and deduplication was also performed. Overall, 654 and 506 reports mentioning respectively ceftolozane-tazobactam and ceftazidime-avibactam were found, with DMEs accounting respectively for 13.1% and 10.9% of cases. *Agranulocytosis* (*N* = 12; LL95% CI = 12.40) and *pancytopenia* (14; 6.18) emerged as unexpected AEs with ceftolozane-tazobactam, while *acute pancreatitis* (7; 8.63) was an over-reported unexpected DME with ceftazidime-avibactam. After deduplication, four unequivocally different cases of *agranulocytosis* with ceftolozane-tazobactam were retained, occurring on average after 8.8 days. Causality was *probable* and *possible* respectively in three and one case. Among neurological AEs exhibiting significant disproportionality, *encephalopathy* with both antibiotics and *mental status changes* with ceftazidime-avibactam were retained in at least three cases after deduplication. Although rare, clinicians should monitor high-risk patients (i.e. individuals affected by haematological malignances, HIV infection, or treated with concomitant myelotoxic agents) for early unexpected occurrence of agranulocytosis with ceftolozane-tazobactam.

**Supplementary Information:**

The online version contains supplementary material available at 10.1007/s10096-020-04149-3.

## Introduction

Ceftolozane-tazobactam (C/T) and ceftazidime-avibactam (C/A) are novel beta-lactam/beta-lactamase inhibitors (BL/BLIs), respectively approved by the Food and Drug Administration (FDA) in 2014 and in 2015, and by the European Medicines Agency (EMA) in 2015 and in 2016, characterized by activity against multidrug-resistant (MDR) Gram-negative pathogens, including extensively drug-resistant *Pseudomonas aeruginosa* (XDR-PA) and carbapenemase producing *Enterobacteriaceae* (CPE) [1].

Although the risk-benefit ratio is generally favourable in severe MDR infections, the safety profile of these antibiotics still requires thorough investigation. Specifically, no differences were found in serious adverse events (AEs) between novel BL/BLIs and controlled treatments in pivotal trials [2], being tolerability predictable and comparable to those seen with other beta-lactams, while real-world evidence is limited to the description of AEs reported with C/A [3]. However, given their expanding use at higher dosage in challenging scenarios, including patients affected by severe renal impairment [4], characterization of potential unexpected AEs with novel BL/BLIs becomes necessary.

The FDA Adverse Event Reporting System (FAERS) has attracted considerable interest among clinicians for accurate and timely characterization of drug-related risks occurring in real-world patients, also including the assessment of antimicrobials in challenging settings [5–7]. These post-marketing studies are particularly suited to early detect rare, unexpected and delayed AEs, which cannot be fully appreciated in pivotal trials, and are recommended for real-time safety assessment of recently marketed drugs.

We queried the FAERS database to characterize AEs of clinical interest reported with novel BL/BLIs, as an aid in prioritizing monitoring in patients affected by severe MDR Gram-negative infections.

## Methods

An observational, retrospective disproportionality analysis was performed to highlight and characterize AEs of clinical interest with higher-than-expected reporting. The FAERS database (public dashboard), the US repository of AEs and medication errors comprising more than 20 million reports gathered worldwide, was queried to retrieve C/T and C/A reports recorded between the first quarter (Q1) of 2015 and Q2 of 2020.

In order to assign a clinical priority to emerging safety issues, the public list developed by the EMA including 62 different reactions was used to select designated medical events (DMEs), namely rare, serious AEs with a recognized drug-attributable risk, which may constitute a safety issue under certain circumstances (e.g. plausible causality with exclusion of alternative causes) [8].

Furthermore, given the non-negligible risk of neurotoxicity reported with cephalosporins [9], we searched clinical signs/symptoms, known as preferred terms (PTs) according to the Medical Dictionary for Regulatory Activities, in the following High Level Group Terms: “Seizures” (comprising 77 PTs), “Deliria” and “Hallucinations” (12 PTs each). Specific PTs concerning neurotoxicity (namely “encephalopathy”, “tremor”, “agitation”, “anxiety”, “cognitive disorder”, “mental impairment”, “altered state of consciousness”, “mental disorder”, “mental status changes”, “myoclonus”, “neurotoxicity”) were also analysed.

The reporting odds ratio (ROR) with relevant 95% confidence interval (CI) was calculated as a measure of disproportionality. All other drugs/events recorded in FAERS and cephalosporins showing clinical evidence of neurological AEs (namely cefazolin, ceftriaxone, cefixime, cefotiam, ceftazidime and cefepime according to the review of Sutter et al. [10]) were respectively selected as comparator for analysis of DMEs and neurotoxicity. Specifically, a case‑non-case approach was applied: Cases were defined by reports of the event of interest for C/T or C/A in which the drug was suspiciously recorded, while non-cases were represented by AE reports recorded for comparators. The ROR is the odds of exposure to C/T or C/A among the cases divided by the odds of exposure to C/T or C/A among the non-cases. If the proportion of the event of interest is greater in patients exposed to C/T or C/A (cases) than in patients exposed to all other drugs reported in FAERS, or for the selected cephalosporins (non-cases), a disproportionality signal emerges. Cases counted as many-fold as the number of DMEs or “neurological” events recorded in a given report. Traditional criteria for signal detections were used, i.e. lower limit of the 95% CI of the ROR > 1 with at least three cases of interest reported [11].

DMEs or neurological AEs emerging from disproportionality analysis were further scrutinized to better describe relevant clinical features: demographic information, reported indication, proportion of death, proportion of septic shock and/or multi-organ dysfunction syndrome and concomitant risk factors and/or drugs implicated in the events of interest (i.e. proportion of renal impairment or underlying nervous abnormalities for neurological events).

DMEs with disproportionality signal were also classified into three broad categories, according to the predictability of the reaction: (1) expected AEs, anticipated from pre-marketing pivotal trials; (2) disease-related AEs, for which underlying sepsis/septic shock represents per se a risk factor; and (3) unexpected AEs, on the basis of pharmacodynamic properties.

Unexpected DMEs and neurological AEs were further scrutinized to detect potential duplicates, based on overlapping data in six key fields, as previously performed [6]: event date, age, sex, reporter’s country, concomitant reactions and concomitant drugs. Records with at least five out of six overlaps were considered duplicates and excluded by case assessment.

Finally, unexpected DMEs with at least 3 unduplicated cases, a widely accepted signalling criterion [11], were further characterized by accessing original narratives submitted by the reporter through a Freedom of Information Act requested to the FDA: Information on medical history, laboratory findings, dechallenge/rechallenge, medical management and latency were used to assess causality according to the WHO system [12].

## Results

Overall, 654 and 506 reports mentioning respectively C/T and C/A as suspect were found, of which 65.4% and 87.2% were serious. Subjects aged > 50 years old were the most represented, with slight male preponderance for both C/T and C/A (Supplementary Table 1).

DMEs were respectively reported in 86 (13.1%) and 55 cases (10.9%) with C/T and C/A; 12 and 13 DMEs were reported at least once, respectively. Disproportionality analysis was performed for 4 and 6 DMEs respectively with C/T and C/A (eight and seven AEs were reported in less than three cases; Supplementary Table 2). Increased reporting was found for *acute kidney injury* (*N* = 24; ROR 5.50; 95% CI 3.66–8.27), *agranulocytosis* (12; 21.96; 12.40–38.87), *pancytopenia* (14; 10.50; 6.18–17.82) and *renal failure* (27; 7.88; 5.36–11.58) with C/T. *Acute kidney injury* (16; 4.71; 2.86–7.76), *anaphylactic shock* (3; 6.85; 2.20–21.33), *haemolytic anaemia* (3; 11.56; 3.72–35.98), *hepatic failure* (4; 5.74; 2.15–15.36), *acute pancreatitis* (7; 18.19; 8.63–38.36) and *renal failure* (13; 4.82; 2.78–8.37) emerged as over-reported DMEs with C/A (Table 1).

**Table 1:** Designated medical events (DMEs) and selected neurological adverse events (AEs) reported with ceftolozane-tazobactam and ceftazidime-avibactam showing statistically significant disproportionality. Main clinical features after deduplication are also showed

DMEs or neurological AEs	No. of cases	ROR (95% CI)	Predictability	No. of cases after deduplication	Proportion of potential confounders*
Ceftolozane-tazobactam – DMEs					
Acute kidney injury	24	5.50 (3.66–8.27)	Expected Disease-related	10	5 (50.0%)
Agranulocytosis	12	21.96 (12.40–38.87)	Unexpected	4	1 (25.0%)
Pancytopenia	14	10.50 (6.18–17.82)	Unexpected	2	1 (50.0%)
Renal failure	27	7.88 (5.36–11.58)	Expected Disease-related	12	6 (50.0%)
Ceftazidime-avibactam – DMEs					
Acute kidney injury	16	4.71 (2.86–7.76)	Expected Disease-related	9	4 (44.4%)
Anaphylactic shock	3	6.85 (2.20–21.33)	Expected	2	0 (0.0%)
Haemolytic anaemia	3	11.56 (3.72–35.98)	Expected	3	0 (0.0%)
Hepatic failure	4	5.74 (2.15–15.36)	Disease-related	3	0 (0.0%)
Pancreatitis acute	7	18.19 (8.63–38.36)	Unexpected	2	2 (100.0%)
Renal failure	13	4.82 (2.78–8.37)	Expected Disease-related	8	6 (75.0%)
Ceftolozane-tazobactam – selected neurological AEs					
Encephalopathy	19	2.63 (1.66–4.19)	Expected	3	1 (33.3%)
Epilepsy	11	3.61 (1.96–6.65)	Expected	1	0 (0.0%)
Generalized tonic-clonic seizure	10	6.60 (3.43–12.70)	Expected	2	0 (0.0%)
Status epilepticus	10	1.93 (1.03–3.65)	Expected	1	0 (0.0%)
Ceftazidime-avibactam – selected neurological AEs					
Encephalopathy	18	3.25 (2.01–5.24)	Expected	10	3 (30.0%)
Mental status changes	8	4.04 (1.98–8.26)	Expected	7	3 (42.9%)
Tonic convulsion	4	14.42 (4.99–41.71)	Expected	1	0 (0.0%)

*AEs* adverse events; *DMEs* designated medical events; *ROR* reporting odds ratio; *CI* confidence interval

*Concomitant drugs or underlying conditions potentially implicated in the specific adverse event

*Agranulocytosis* and *pancytopenia* with C/T and *pancreatitis acute* with C/A emerged as unexpected AEs. Overall, concomitant drugs or underlying conditions potentially confounding were retrieved in 45.5% of cases (Supplementary Table 3).

After deduplication, only two cases of *pancytopenia* with C/T and two cases of *pancreatitis acute* with C/A were found as unequivocally different. Narratives were requested for the four confirmed cases of *agranulocytosis* with C/T (Table 2). Patients were ≥ 70 years old with female preponderance (75.0%). Agranulocytosis occurred after an average of 8.8 days (range 4–17 days) following the beginning of C/T treatment. In two cases, potential confounders related to underlying conditions (i.e. HIV infection, acute leukaemia, lymphoma) or concomitant therapies (i.e. rituximab, allopurinol, trimethoprim-sulfamethoxazole, metimazole) were recorded. In three cases, C/T was withdrawn, and in two patients, the administration of granulocyte colony-stimulating factor was required. All events were serious, leading to prolonged hospitalization. Recovery and increased in neutrophil count occurred in all cases after approximately 5–10 days. Causality was *probable* in three cases and *possible* in one case.

**Table 2:** Narrative review of cases of agranulocytosis reported with ceftolozane-tazobactam and submitted to FAERS

Case ID	Age/sex	Reporter country	Dose/route of administration	Time onset (days)*	Medical history	Concomitant therapies	Acute medical condition	Laboratory and imaging findings	Specific treatment	Dechallenge/rechallenge	Outcome	Causality assessment
13844290	87/M	Spain	1 g q8h IV	17	High blood pressure, congestive heart failure, atrial fibrillation, chronic obstructive pulmonary disease (COPD) with oxygen therapy, insomnia, loss of personal independence in daily activities, arthralgia	Amiodarone 200 mg/day Furosemide 40 mg q12h Trazodone 100 mg/day Tramadol 50 mg q8h	COPD decompensation due to LRTI caused by PA XDR	Neutrophil count: 2.96 × 10^9^/L before treatment with C/T 1.33 × 10^9^/L at the moment of C/T withdrawn 5.45 × 10^9^/L at recovering	C/T withdrawn 1 day after the occurrence of agranulocytosis Progressive increase in leucocyte count after C/T withdrawn	Yes/No	Prolonged hospitalization Recovered after 6 days	Probable (reasonable time relationship with drug intake; unlikely role of other diseases/drugs; positive dechallenge)
14034547	79/F	France	LD 1 g IV MD 250 mg q8h IV Undergoing haemodialysis	8	Atrial fibrillation, pulmonary embolism	Amiodarone Fluindione	cUTI with bacteraemia caused by PA MDR	Before treatment with C/T Neutrophil count: 1.76 G/L White cell count: 2.84 G/L During C/T treatment Neutrophil count: 0.13 G/L White cell count: 2.61 G/L Osteomedullar biopsy: decrease of the granulocytic density at 10%, no impairment of other cell lines. Suspected toxic cause. At recovering Neutrophil count: 6.13 G/L White cell count: 8.29 G/L	C/T withdrawn 1 day after occurrence of agranulocytosis Switch to ceftazidime-avibactam and administration of filgrastim Progressive increase in leucocytes and neutrophil count after C/T withdrawn	Yes/No	Prolonged hospitalization Recovered	Probable (osteomedullar biopsy suggestive for toxic cause; reasonable time relationship with drug intake; unlikely role of other diseases/drugs; positive dechallenge)
15505937	71/F	Portugal	3 g q8h IV	4	NHL with medullar invasion Mature B cell type acute leukaemia undergoing induction chemotherapy Controlled HIV infection	Fluconazole 200 mg/day Tigecycline 100 mg q12h Metamizole** Rituximab** TMP-SMX** Allopurinol**	Pneumonia caused by XDR PA	Repeated myelogram: no presence of lymphoblast	C/T continuation Administration of growth factors with no benefit	No/No	Prolonged hospitalization Absolute neutropenia for 10 days with refractoriness to growth factors. Haemoglobin and platelet recovery was maintained. Recovered	Possible (concomitant drugs/diseases potentially explaining the event; dechallenge not performed)
15659483	70/F	Portugal	1.5 g q8h IV	6	HIV infection Depressive symptom Lymphoma Vertigo	Abacavir/Lamivudine 600/300 mg/day Tigecycline 100 mg/day Nevirapine 400 mg/day Allopurinol*** 300 mg/day TMP-SMX*** 800/160 mg q8h	Sepsis	NA	C/T withdrawn 5 days after the occurrence of agranulocytosis	Yes/No	Prolonged hospitalization Life-threatening Recovered/resolved	Probable (reasonable time relationship with drug intake; unlikely role of other diseases/drugs: no worsening of HIV infection; no ongoing treatment for lymphoma; allopurinol and TMP-SMX prescribed 45 days before the event; positive dechallenge)

*COPD* chronic obstructive pulmonary disease; *LRTI* lower tract respiratory infection; *PA Pseudomonas aeruginosa*; *XDR* extensively drug-resistant; *MDR* multidrug-resistant; *C/T* ceftolozane-tazobactam; *cUTI* complicated urinary tract infection; *NHL* non-Hodgkin’s lymphoma; *TMP-SMX* cotrimoxazole; *NA* not available

*After ceftolozane-tazobactam administration

**The indicated drugs were previously administered to the patient before admission without experience of agranulocytosis

***Prescribed 45 days before occurrence of agranulocytosis

Overall, 78 (11.9%) and 71 (14.0%) neurological AEs were respectively found with C/T and C/A. Disproportionality analysis was performed for six and seven AEs respectively with C/T and C/A. Compared to selected cephalosporins, *encephalopathy* (19; 2.63; 1.66–4.19), *epilepsy* (10; 6.60; 3.43–12.70), *generalized tonic-clonic seizure* (11; 3.61; 1.96–6.65) and *status epilepticus* (10; 1.93; 1.03–3.65) reported with C/T exhibited significant ROR. Increased reporting was found for *encephalopathy* (18; 3.25; 2.01–5.24), *mental status changes* (8; 4.04; 1.98–8.26) and *tonic convulsion* (4; 14.42; 4.99–41.71) with C/A (Table 1; Supplementary Table 4).

After deduplication, only *encephalopathy* with both BL and BLIs and *mental status changes* with C/A were confirmed in at least three cases. Overall, concomitant renal impairment was found in 28.0% of cases, while no underlying nervous abnormalities were recognized (Supplementary Table 5).

## Discussion

To the best of our knowledge, this is the first large-scale study reporting serious AEs with novel BL/BLIs. Haematological reactions emerged as unexpected life-threatening AEs for C/T, while C/A exhibited a predictable safety profile according to anticipated common AEs found in pivotal trials (i.e. anaphylactic shock, haemolytic anaemia) and expected clinical complications of severe infections caused by CPE (i.e. septic shock with hepatic/renal failure). Furthermore, an over-reporting of different serious neurological AEs (namely encephalopathy and mental status changes) compared to other cephalosporins [10] in patients receiving C/T or C/A was found.

**Fig. 1:**
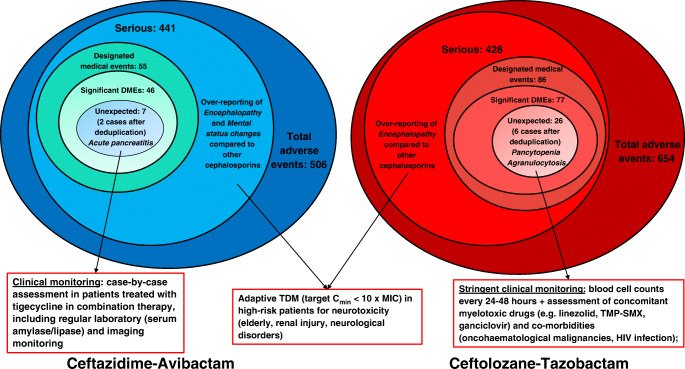
Suggested clinical monitoring for designated medical events (DMEs) and neurological adverse events (AEs) reported with ceftolozane-tazobactam and ceftazidime-avibactam and emerged as significant. TDM: therapeutic drug monitoring; MIC: minimum inhibitory concentration; TMP-SMX: cotrimoxazole

Notably, signals of unexpected pancytopenia and agranulocytosis with C/T, detected by disproportionality, were refuted by our qualitative analysis, including concomitant use of different agents (namely linezolid, ganciclovir, valacyclovir and trimethoprim-sulfamethoxazole) known to cause myelotoxicity. Therefore, possible confounders responsible for synergic toxicity may be implicated in the occurrence of pancytopenia. Conversely, in the four retained cases of agranulocytosis with C/T, the limited presence of concomitant risk factors together with causality assessment suggests a true safety issue. Although currently no case reports or preclinical evidence of ceftolozane-induced agranulocytosis exist, the immune-mediated hypothesis may reasonably explain the occurrence of this unexpected AE with C/T, as reported for other agents, including beta-lactams [13, 14]. However, a dose-dependent direct toxic effect on granulocytopoiesis cannot be excluded [15]. Additionally, elderly and female gender are recognized risk factors for drug-induced agranulocytosis [13]. Notably, mean age of our cases was approximately 77 years with female preponderance (75.0%). Furthermore, the mean onset time of our cases (8.8 days) overlaps with agranulocytosis reported in 12 patients with piperacillin-tazobactam (17.6 days) and in 62 subjects treated with different beta-lactams (16 days) [14, 15], consistently with the immune-mediated hypothesis. Although in two of our cases potential confounders (underlying diseases and/or concomitant myelotoxic drugs) were found, all these agents were previously administered for several cycles without occurrence of agranulocytosis, nor was any previous event of neutropenia noted in patients affected by leukaemia or lymphoma. Notably, in three cases, a probable causal association was detected, and none was classified as unlikely. Given the non-negligible proportion of XDR-PA infections in haematological patients [16], C/T may assume a leading role in this scenario. Consequently, the over-reporting of agranulocytosis calls for clinical monitoring in patients treated with C/T. Particularly, assessment of host-dependent risk factors (i.e. elderly, haematological malignances, HIV infection, concomitant myelotoxic agents) coupled with intensive blood cell count monitoring (every 24 h during the entire treatment with C/T) should be implemented (Fig. 1).

The two unduplicated cases of acute pancreatitis with C/A deserve attention for the following reasons: In the literature, no case of definite or probable association was found for ceftazidime [17]; in our reports, concomitant use of tigecycline, known to cause pancreatitis [18], was noted. Although C/A could not be considered the only primary suspect, considering its wide use in combination with tigecycline in complicated intraabdominal infections caused by CPE [19], clinicians should evaluate the risk of pancreatitis on a case-by-case basis in critically ill patients treated with drug combination, possibly implementing additional laboratory (i.e. serum amylase/lipase) or imaging monitoring (Fig. 1).

Our analysis found an over-reporting of different serious neurological AEs with novel BL/BLIs compared to selected cephalosporins. Although neurotoxicity is a well-known safety concern in susceptible patients with risk factors (i.e. elderly, renal impairment, underlying nervous abnormalities) [9, 10], the higher reporting with novel BL/BLIs, rather than reflecting an intrinsically higher risk related to these agents per se, may be the result of a channelling bias favoured by their well-established use at standard/higher dosage to improve efficacy in severe MDR infections in critical settings [4]. To this regard, although C/A includes a maximum dose of ceftazidime (namely 6 g/day) for subject with normal renal function, novel BL/BLIs are commonly used in real-world scenarios at higher than recommended dosage also in patients with severe renal impairment or requiring continuous renal replacement therapy (CRRT), in order to overcome pharmacokinetic issues usually observed in critically ill subjects (e.g. wide increase in volume of distribution, high intensity CRRT, prompt recovery of renal function in the first 48–72 h), and achieve aggressive PK/PD targets directly associated with better clinical outcome and prevention of resistance development [4, 20–22]. However, in this scenario, it could be possible that toxic serum concentrations are more likely achieved, thus leading to a greater risk of neurological AEs. Adaptive therapeutic drug monitoring of novel BL/BLIs could be useful, retaining steady-state concentrations below tenfold the minimum inhibitory concentration (Fig. 1) [23].

Notwithstanding the well-known limitations of our approach (e.g. potential reporting biases, including under-reporting phenomenon, lack of exposure data and clinical details, inability in establishing firm causality between drug exposure and occurrence of AEs, possible remaining duplicates), agranulocytosis emerged as an early and unexpected, albeit rare, AEs with C/T, thus calling for both stringent clinical monitoring in high-risk patients (namely those affected by haematological malignancies, HIV infection, acute or chronic kidney injury, or treated with concomitant myelotoxic or nephrotoxic agents) affected by severe MDR Gram-negative infections, and further observational studies to better characterize this safety aspect.

## Supplementary information

ESM 1 (DOCX 57 kb).

ESM 2 (XLSX 120 kb).

ESM 3 (XLSX 95 kb).

## Data Availability

Data supporting the findings of this study were derived from the following resource available in the public domain: https://www.fda.gov/drugs/questions-and-answers-fdas-adverse-event-reporting-system-faers/fda-adverse-event-reporting-system-faers-public-dashboard.
