# Supplementary material for: Serious adverse events with novel beta-lactam/beta-lactamase inhibitor combinations: a large-scale pharmacovigilance analysis
Source: Eur J Clin Microbiol Infect Dis. 2021 Jan 7;40(6):1169–76. doi: 10.1007/s10096-020-04149-3 (PMC8139903; doi:10.1007/s10096-020-04149-3)
Supplement: Supplementary file 1 — (DOCX 57 kb). [file 10096_2020_4149_MOESM1_ESM.docx]

**Supplementary materials**

**Supplementary Table 1 –** Demographic features of adverse events reported in the FAERS database with ceftolozane-tazobactam and ceftazidime-avibactam

**Supplementary Table 2 –** Overall designated medical events (DMEs) recorded with ceftolozane-tazobactam and ceftazidime-avibactam. Reporting odds ratios (RORs) were calculated only for DMEs with at least three cases.

**Supplementary Table 3 –** Case-by-case assessment of designated medical events (DMEs) reported with ceftolozane-tazobactam and ceftazidime-avibactam showing statistically significant disproportionality. Only cases retained after deduplication are included.

**Supplementary Table 4 –** Overall selected neurological adverse events (AEs) recorded with ceftolozane-tazobactam and ceftazidime-avibactam. Reporting odds ratios (RORs) were calculated only for AEs with at least three cases.

**Supplementary Table 5 -** Case-by-case assessment of neurological adverse events reported with ceftolozane-tazobactam and ceftazidime-avibactam showing statistically significant disproportionality. Only cases retained after deduplication were included.

**Supplementary table 1 –** Demographic features of adverse events reported in the FAERS database with ceftolozane-tazobactam and ceftazidime-avibactam

| **Demographic features** | **Ceftolozane-Tazobactam** | **Ceftazidime-Avibactam** |
| --- | --- | --- |
| *Overall number of cases* | 654 | 506 |
| *Sex*  Female  Male  Not specified | 194 (29.7%)  283 (43.3%)  177 (27.0%) | 163 (32.2%)  196 (38.7%)  147 (29.1%) |
| *Age (years)*  < 10  10-19  20-29  30-39  40-49  50-59  60-69  70-79  ≥ 80  Not specified | 14 (2.1%)  19 (2.9%)  36 (5.5%)  52 (8.0%)  43 (6.6%)  58 (8.9%)  62 (9.5%)  93 (14.2%)  27 (4.1%)  250 (38.2%) | 8 (1.6%)  10 (2.0%)  21 (4.1%)  15 (3.0%)  17 (3.4%)  44 (8.7%)  53 (10.5%)  39 (7.7%)  21 (4.1%)  278 (54.9%) |
| *Reporter country*  Europe  North America  South America  Asia  Africa  Oceania  Not specified | 272 (41.6%)  330 (50.5%)  15 (2.3%)  29 (4.4%)  0 (0.0%)  3 (0.5%)  5 (0.8%) | 289 (57.1%)  116 (22.9%)  28 (5.5%)  58 (11.5%)  1 (0.2%)  1 (0.2%)  13 (2.6%) |
| *Seriousness*  Serious  Non-serious | 428 (65.4%)  226 (34.6%) | 441 (87.2%)  65 (12.8%) |
| *Outcome**  Congenital anomaly  Death  Disability  Hospitalization  Life-threatening  Other outcomes | 0 (0.0%)  142 (21.7%)  0 (0.0%)  191 (29.2%)  32 (4.9%)  296 (45.3%) | 0 (0.0%)  211 (41.7%)  0 (0.0%)  120 (23.7%)  31 (6.1%)  226 (44.7%) |
| *Reporter type*  Consumer  Healthcare professionals  Missing | 18 (2.8%)  635 (97.1%)  1 (0.1%) | 24 (4.7%)  481 (95.1%)  1 (0.2%) |

* one case may exhibit more than one outcome

**Supplementary Table 2 –** Overall designated medical events (DMEs) recorded with ceftolozane-tazobactam and ceftazidime-avibactam. Reporting odds ratios (RORs) were calculated only for DMEs showing at least three cases.

| **DMEs** | **Ceftolozane-Tazobactam** | | **Ceftazidime-Avibactam** | |
| --- | --- | --- | --- | --- |
|  | **No. cases** | **ROR (95% CI)** | **No. cases** | **ROR (95% CI)** |
| Acute hepatic failure | 1 | - | 1 | - |
| Acute kidney injury | 24 | 5.50 (3.66-8.27) | 16 | 4.71 (2.86-7.76) |
| Agranulocytosis | 12 | 21.96 (12.40-38.87) | 1 | - |
| Anaphylactic reaction | 0 | - | 1 | - |
| Anaphylactic shock | 0 | - | 3 | 6.85 (2.20-21.33) |
| Anaphylactoid reaction | 0 | - | 0 | - |
| Anaphylactoid shock | 0 | - | 0 | - |
| Angioedema | 1 | - | 0 | - |
| Aplasia pure red cell | 0 | - | 0 | - |
| Aplastic anaemia | 0 | - | 0 | - |
| Autoimmune haemolytic anaemia | 0 | - | 0 | - |
| Autoimmune hepatitis | 0 | - | 0 | - |
| Autoimmune pancreatitis | 0 | - | 0 | - |
| Azotaemia | 0 | - | 0 | - |
| Blindness | 1 | - | 0 | - |
| Bone marrow failure | 1 | - | 0 | - |
| Deafness | 0 | - | 0 | - |
| Deafness neurosensory | 0 | - | 0 | - |
| Deafness permanent | 0 | - | 0 | - |
| Deafness transitory | 0 | - | 0 | - |
| Dermatitis exfoliative | 0 | - | 0 | - |
| Dermatitis exfoliative generalised | 0 | - | 2 | - |
| Drug reaction with eosinophilia and systemic symptoms | 1 | - | 1 | - |
| Drug-induced liver injury | 0 | - | 1 | - |
| Erythema multiforme | 1 | - | 0 | - |
| Febrile neutropenia | 0 | - | 0 | - |
| Granulocytopenia | 0 | - | 0 | - |
| Haemolysis | 0 | - | 0 | - |
| Haemolytic anaemia | 0 | - | 3 | 11.56 (3.72-35.98) |
| Hepatic failure | 2 | - | 4 | 5.74 (2.15-15.36) |
| Hepatic infarction | 0 | - | 0 | - |
| Hepatic necrosis | 0 | - | 0 | - |
| Hepatitis fulminant | 0 | - | 0 | - |
| Immune thrombocytopenic purpura | 0 | - | 0 | - |
| Intestinal perforation | 0 | - | 0 | - |
| Ischaemic pancreatitis | 0 | - | 0 | - |
| Neutropenic colitis | 0 | - | 0 | - |
| Neutropenic infection | 0 | - | 0 | - |
| Neutropenic sepsis | 0 | - | 0 | - |
| Oedematous pancreatitis | 0 | - | 0 | - |
| Optic ischaemic neuropathy | 0 | - | 0 | - |
| Pancreatitis | 0 | - | 0 | - |
| Pancreatitis acute | 1 | - | 7 | 18.19 (8.63-38.36) |
| Pancytopenia | 14 | 10.50 (6.18-17.82) | 0 | - |
| Product contamination microbial | 0 | - | 0 | - |
| Progressive multifocal leukoencephalopathy | 0 | - | 0 | - |
| Pulmonary arterial hypertension | 0 | - | 0 | - |
| Pulmonary fibrosis | 0 | - | 0 | - |
| Pulmonary hypertension | 0 | - | 0 | - |
| Renal failure | 27 | 7.88 (5.36-11.58) | 13 | 4.82 (2.78-8.37) |
| Reye's syndrome | 0 | - | 0 | - |
| Rhabdomyolysis | 0 | - | 0 | - |
| Stevens-Johnson syndrome | 0 | - | 0 | - |
| Sudden cardiac death | 0 | - | 0 | - |
| Sudden hearing loss | 0 | - | 0 | - |
| Sudden visual loss | 0 | - | 0 | - |
| Thrombotic thrombocytopenic purpura | 0 | - | 0 | - |
| Torsade de pointes | 0 | - | 0 | - |
| Toxic epidermal necrolysis | 0 | - | 2 | - |
| Toxic optic neuropathy | 0 | - | 0 | - |
| Transmission of an infectious agent via product | 0 | - | 0 | - |
| Ventricular fibrillation | 0 | - | 0 | - |

DME, designated medical event; ROR, reporting odds ratio; CI, confidence interval.

**Supplementary Table 3 –** Case-by-case assessment of designated medical events (DMEs) reported with ceftolozane-tazobactam and ceftazidime-avibactam showing statistically significant disproportionality. Only cases retained after deduplication are included.

| **AE** | **No. cases** | **No. deaths** | **Proportion of death** | **Mean age** | **Sex** | **Reported indication** | **Proportion of septic shock / MODS** | **Proportion of potential confounders**  **(concomitant drugs or underlying conditions)** | **Predictability** |
| --- | --- | --- | --- | --- | --- | --- | --- | --- | --- |
| ***Ceftolozane-Tazobactam*** | | | | | | | | | |
| **Acute kidney injury** | 10 | 1 | 10.0% | 56.3 ± 14.1  (1 NS) | 8 M – 2 F | 3 Bacteraemia *Pseudomonas*  2 Pneumonia *Pseudomonas*  1 *Pseudomonas* infection  1 Device-related infection  1 Urinary tract infection *Escherichia*  1 Infection  1 Septic shock | 4 (40.0%) | 5 (50.0%)  2 Gentamicin  2 Colistin  1 Amikacin  1 Vancomycin  1 Tobramycin | Expected  Disease-related |
| **Agranulocytosis** | 4 | 0 | 0.0% | 76.8 ± 7.9 | 1 M – 3 F | 1 Pneumonia *Pseudomonas*  1 *Pseudomonas* infection  1 Bacteraemia  1 Sepsis | 0 (0.0%) | 1 (25.0%)  1 HIV infection with lymphoma  1 TMP/SMX | Unexpected |
| **Pancytopenia** | 2 | 0 | 0.0% | 43.0  (1 NS) | 1 M – 1 F | 1 Cellulitis  1 NS | 0 (0.0%) | 1 (50.0%)  1 Valacyclovir  1 TMP/SMX  1 Linezolid  1 Ganciclovir | Unexpected |
| **Renal failure** | 12 | 5 | 41.7% | 59.3 ± 14.1  (4 NS) | 5 M – 5 F  2 NS | 4 NS  2 Peritonitis  2 Pneumonia  1 Abdominal infection  1 Sepsis *Pseudomonas*  1 Pneumonia *Pseudomonas*  1 Osteomyelitis *Pseudomonas*  1 Renal abscess | 5 (41.7%) | 6 (50.0%)  3 Vancomycin  3 Colistin  2 Tobramycin  1 Amikacin | Expected  Disease-related |
| ***Ceftazidime-Avibactam*** | | | | | | | | | |
| **Acute kidney injury** | 9 | 3 | 33.3% | 64.5 ± 5.3  (3 NS) | 3 M – 5 F  1 NS | 2 Sepsis  2 Urinary tract infection  1 Pneumonia  1 *Klebsiella* infection  1 *Pseudomonas* infection  1 *Enetrobacter* infection  1 NS | 1 (11.1%) | 4 (44.4%)  2 Colistin  1 Vancomycin  1 Amikacin | Expected  Disease-related |
| **Anaphylactic shock** | 2 | 1 | 50.0% | 92.0  (1 NS) | 1 M – 1 F | 1 Pneumonia *Klebsiella*  1 Bacteraemia  1 NS | 1 (50.0%) | 0 (0.0%) | Expected |
| **Haemolytic anaemia** | 3 | 0 | 0.0% | 67.0  (2 NS) | 0 M – 3 F | 2 Pneumonia *Klebsiella*  1 Wound infection *Klebsiella* | 0 (0.0%) | 0 (0.0%) | Expected |
| **Hepatic failure** | 3 | 3 | 100.0% | 77.0  (2 NS) | 3 M | 1 Bacteraemia *Klebsiella*  1 Pneumonia *Klebsiella*  1 Peritonitis | 3 (100.0%) | 0 (0.0%) | Disease-related |
| **Pancreatitis acute** | 2 | 1 | 50.0% | 58.0 ± 5.7 | 2 F | 1 Lung abscess  1 Pneumonia *Klebsiella* | 1 (50.0%) | 2 (100.0%)  2 Tigecycline | Unexpected |
| **Renal failure** | 8 | 4 | 50.0% | 66.8 ± 22.2  (4 NS) | 6 M – 1 F  1 NS | 2 Infections  2 Pneumonia *Klebsiella*  1 Bacteraemia *Klebsiella*  1 Peritonitis  1 Urinary tract infection  1 *Burkholderia Cepacia* complex infection in cystic fibrosis | 4 (50.0%) | 6 (75.0%)  3 Furosemide  2 Vancomycin  2 Colistin  2 Gentamicin  1 Amikacin | Expected  Disease-related |

ROR: reporting odds ratio; CI: confidence interval; NS: not specified; AEs: adverse events; DMEs: designated medical events; MODS: multi-organ dysfunction syndrome; TMP/SMX: cotrimoxazole

**Supplementary Table 4 –** Overall selected neurological adverse events (AEs) recorded with ceftolozane-tazobactam and ceftazidime-avibactam. Reporting odds ratios (RORs) were calculated only for AEs showing at least three cases.

| **Neurological AEs** | **Ceftolozane-Tazobactam** | | **Ceftazidime-Avibactam** | |
| --- | --- | --- | --- | --- |
|  | **No. cases** | **ROR (95% CI)** | **No. cases** | **ROR (95% CI)** |
| Encephalopathy | 19 | 2.63 (1.66-4.19) | 18 | 3.25 (2.01-5.24) |
| Tremor | 1 | - | 1 | - |
| Agitation | 0 | - | 1 | - |
| Anxiety | 0 | - | 1 | - |
| Cognitive disorder | 1 | - | 0 | - |
| Epileptic encephalopaty | 0 | - | 0 | - |
| Mental impairment | 0 | - | 1 | - |
| Altered state of consciousness | 1 | - | 1 | - |
| Mental disorder | 0 | - | 1 | - |
| Mental status changes | 0 | - | 8 | 4.04 (1.98-8.26) |
| Myoclonus | 1 | - | 5 | 1.88 (0.77-4.57) |
| Neurotoxicity | 1 | - | 4 | 1.91 (0.70-5.15) |
| CAR T-cell-related encephalopathy syndrome | 0 | - | 0 | - |
| Confusion postoperative | 0 | - | 0 | - |
| Confusional state | 11 | 0.96 (0.53-1.75) | 2 | - |
| Disorentation | 0 | - | 0 | - |
| Postictal state | 0 | - | 0 | - |
| Preictal state | 0 | - | 0 | - |
| Delirium | 1 | - | 2 | - |
| Delirium febrile | 0 | - | 0 | - |
| Delirium tremens | 0 | - | 0 | - |
| Intensive care unit delirium | 0 | - | 0 | - |
| Post-injection delirium sedation syndrome | 0 | - | 0 | - |
| Postoperative delirium | 0 | - | 0 | - |
| Charles Bonnet syndrome | 0 | - | 0 | - |
| Formication | 0 | - | 0 | - |
| Hallucination | 0 | - | 0 | - |
| Hallucination auditory | 0 | - | 0 | - |
| Hallucination olfactory | 0 | - | 0 | - |
| Hallucination gustatory | 0 | - | 0 | - |
| Hallucination synaesthetic | 0 | - | 0 | - |
| Hallucination tactile | 0 | - | 0 | - |
| Hallucination visual | 0 | - | 0 | - |
| Hallucinations mixed | 0 | - | 0 | - |
| Paroxysmal perceptual alteration | 0 | - | 0 | - |
| Somatic hallucination | 0 | - | 0 | - |
| Generalised non-convulsive epilepsy | 0 | - | 0 | - |
| Petit mal epilepsy | 0 | - | 0 | - |
| Benign familial neonatal convulsions | 0 | - | 0 | - |
| Generalised tonic-clonic seizure | 11 | 3.61 (1.96-6.65) | 1 | - |
| Automatism epileptic | 0 | - | 0 | - |
| Dreamy state | 0 | - | 0 | - |
| Epileptic psychosis | 0 | - | 0 | - |
| Focal dyscognitive seizures | 1 | - | 0 | - |
| Temporal lobe epilepsy | 0 | - | 0 | - |
| Transient epileptic amnesia | 0 | - | 0 | - |
| Uncinate fits | 0 | - | 0 | - |
| Autonomic seizure | 0 | - | 0 | - |
| Deja vu | 0 | - | 0 | - |
| Simple partial seizure | 0 | - | 0 | - |
| Acquired epileptic aphasia | 0 | - | 0 | - |
| Acute encephalitis with refractory, repetitive partial seizure | 0 | - | 0 | - |
| Alcoholic seizure | 0 | - | 0 | - |
| Atonic seizures | 0 | - | 0 | - |
| Atypical benign partial epilepsy | 0 | - | 0 | - |
| Baltic myoclonic epilepsy | 0 | - | 0 | - |
| Benign rolandic epilepsy | 0 | - | 0 | - |
| Biotinidase deficiency | 0 | - | 0 | - |
| Change in seizure presentation | 0 | - | 0 | - |
| Citrate transporter deficiency | 0 | - | 0 | - |
| Clonic convulsion | 0 | - | 1 | - |
| Convulsion in childhood | 0 | - | 0 | - |
| Convulsions local | 0 | - | 0 | - |
| Convulsive threshold lowered | 0 | - | 0 | - |
| CSWS syndrome | 0 | - | 0 | - |
| Drug withdrawal convulsions | 0 | - | 0 | - |
| Early infantile epileptic encephalopathy with burst-suppression | 0 | - | 0 | - |
| Eclampsia | 0 | - | 0 | - |
| Epilepsy | 10 | 6.60 (3.43-12.70) | 1 | - |
| Epilepsy congenital | 0 | - | 0 | - |
| Epilepsy with myoclonic-atonic seizures | 0 | - | 0 | - |
| Epileptic aura | 0 | - | 0 | - |
| Epileptic encephalopathy | 0 | - | 0 | - |
| Eyelid myoclonus | 0 | - | 0 | - |
| Febrile convulsion | 0 | - | 0 | - |
| Febrile infection-related epilepsy syndrome | 0 | - | 0 | - |
| Frontal lobe epilepsy | 0 | - | 0 | - |
| Hyperglycemic seizure | 0 | - | 0 | - |
| Hypocalcemic seizure | 0 | - | 0 | - |
| Hypoglycemic seizure | 0 | - | 0 | - |
| Hyponatraemic seizure | 0 | - | 0 | - |
| Idiopathic generalized epilepsy | 0 | - | 0 | - |
| Idiopathic partial epilepsy | 0 | - | 0 | - |
| Infantile spasms | 0 | - | 0 | - |
| Isodicentric chromosome 15 syndrome | 0 | - | 0 | - |
| Juvenile myoclonic epilepsy | 0 | - | 0 | - |
| Lafora's myoclonic epilepsy | 0 | - | 0 | - |
| Lennox-Gastaut syndrome | 0 | - | 0 | - |
| Migraine-triggered seizure | 0 | - | 0 | - |
| Molybdenum cofactor deficiency | 0 | - | 0 | - |
| Myoclonic epilepsy | 0 | - | 0 | - |
| Myoclonic epilepsy and ragged-red fibers | 0 | - | 0 | - |
| Neonatal epileptic seizure | 0 | - | 0 | - |
| Neonatal seizure | 0 | - | 0 | - |
| Partial seizures | 0 | - | 1 | - |
| Partial seizures with secondary generalization | 0 | - | 0 | - |
| Phelan-McDermid syndrome | 0 | - | 0 | - |
| Post stroke epilepsy | 0 | - | 0 | - |
| Post stroke seizure | 0 | - | 0 | - |
| Post-traumatic epilepsy | 0 | - | 0 | - |
| Postictal depression | 0 | - | 0 | - |
| Postictal psychosis | 0 | - | 0 | - |
| Seizure | 10 | 0.89 (0.48-1.67) | 13 | 1.51 (0.87-2.64) |
| Seizure anoxic | 0 | - | 0 | - |
| Seizure cluster | 0 | - | 0 | - |
| Seizure like phenomena | 0 | - | 0 | - |
| Severe myoclonic epilepsy of infancy | 0 | - | 0 | - |
| Status epilepticus | 10 | 1.93 (1.03-3.65) | 5 | 1.24 (0.51-3.02) |
| Sudden unexplained death in epilepsy | 0 | - | 0 | - |
| Tonic clonic movements | 0 | - | 0 | - |
| Tonic convulsion | 0 | - | 4 | 14.42 (4.99-41.71) |
| Tonic posturing | 0 | - | 0 | - |
| Tuberous sclerosis complex | 0 | - | 0 | - |

AE: adverse event; ROR: reporting odds ratio; CI: confidence interval.

**Supplementary Table 5 -** Case-by-case assessment of neurological adverse events reported with ceftolozane-tazobactam and ceftazidime-avibactam showing statistically significant disproportionality. Only cases retained after deduplication were included.

| **AEs** | **No. cases** | **No. deaths** | **Proportion of death** | **Mean age** | **Sex** | **Reported indication** | **Proportion of septic shock / MODS** | **Proportion of potential confounders**  **(concomitant drugs or underlying conditions)** |
| --- | --- | --- | --- | --- | --- | --- | --- | --- |
| ***Ceftolozane-Tazobactam*** | | | | | | | | |
| **Encephalopathy** | 3 | 1 | 33.3% | 57.3 ± 26.3 | 2 F – 1 M | 1 Pneumonia  1 Sepsis  1 Pyelonephritis | 2 (66.7%) | Renal impairment  1 (33.3%)  Underlying nervous abnormalities  0 (0.0%) |
| **Epilepsy** | 1 | 0 | 0.0% | 73.0 | 1 F | 1 Infection | 0 (0.0%) | Renal impairment  0 (0.0%)  Underlying nervous abnormalities  0 (0.0%) |
| **Generalised tonic-clonic seizure** | 2 | 0 | 0.0% | 55.5 ± 24.7 | 1 F – 1 M | 1 Infection  1 Osteomyelitis | 0 (0.0%) | Renal impairment  0 (0.0%)  Underlying nervous abnormalities  0 (0.0%) |
| **Status epilepticus** | 1 | 0 | 0.0% | 73.0 | 1 F | 1 Infection | 0 (0.0%) | Renal impairment  0 (0.0%)  Underlying nervous abnormalities  0 (0.0%) |
| ***Ceftazidime-Avibactam*** | | | | | | | | |
| **Encephalopathy** | 10 | 4 | 40.0% | 58.2 ± 19.0  (5 NS) | 5 F – 5 M | 3 Complicated urinary tract infection  2 Infection  1 Septic shock  1 Cholangitis  1 Pneumonia  1 Pneumonia *Klebsiella*  1 *Pseudomonas* infection | 1 (10.0%) | Renal impairment  3 (30.0%)  Underlying nervous abnormalities  0 (0.0%) |
| **Mental status changes** | 7 | 0 | 0.0% | 63.3 ± 4.6 | 5 F – 2 M | 2 Liver abscess  2 NS  1 Complicated urinary tract infection  1 Cellulitis  1 *Klebsiella* infection  1 *Citrobacter* infection | 2 (28.6%) | Renal impairment  3 (42.9%)  Underlying nervous abnormalities  0 (0.0%) |
| **Tonic convulsion** | 1 | 0 | 0.0% | 63.0 | 1 M | 1 *Pseudomonas* infection | 1 (100.0%) | Renal impairment  0 (0.0%)  Underlying nervous abnormalities  0 (0.0%) |

NS: not specified; AEs: adverse events; MODS: multi-organ dysfunction syndrome
